# Gunshot injuries: experience in a tertiary health facility in the Niger Delta Region of Nigeria

**DOI:** 10.11604/pamj.2022.43.133.31587

**Published:** 2022-11-09

**Authors:** David Odoyoh Odatuwa-Omagbemi, Cletus Ikechukwu Otene, Roy Efetobor Tony Enemudo, Ejiro Segun Imonijevwe, Theophilus Erhigigwe Sefia

**Affiliations:** 1 Department of Surgery, Delta State University, Abraka, Nigeria,; 2 Department of Orthopaedics and Traumatology, Delta State University Teaching Hospital, Oghara, Delta State, Nigeria

**Keywords:** Gunshot, injuries, armed robbery, fractures

## Abstract

**Introduction:**

the incidence of gunshot injuries and its negative socio-economic impact has assumed an alarming dimension in our environment in recent times as a result of increase in various criminal activities arising largely from poverty including armed robbery, insurgencies, banditry, kidnappings, political thuggery and the like. We share our experience in our centre.

**Methods:**

a retrospective study of cases of gunshot injuries seen, admitted and managed at our health facility over a three-year period. Relevant information including: biodata, circumstances surrounding shooting, type/caliber of gun used, etc. were obtained from patient's case notes and other sources. Data were analysed using SPSS version 18.

**Results:**

forty-one gunshot injury patients made up of 37 males and 4 females were studied. About 68% of the patients fell within the age group of 20-39 years. Students were the most commonly affected group (21%). Armed robbery was the most common aetiology of GSI in our patients (43.9%). Low-velocity guns were largely used (46%). The extremities were the most commonly injured (65.9%). Fractures occurred in 63.4% of patients the femur being the most frequently fractured (22.6%). Patients received various treatment modalities including, wound debridement (78%) and exploratory laparotomies (26.8%) while 3 (7.35%) of them died.

**Conclusion:**

armed robbery and other criminal activities continue to constitute important factors responsible for GSI in our environment. There is need for government and all stakeholders to do more in terms of fighting crime in addition to placing policies to alleviate socioeconomic deprivation.

## Introduction

Firearms violence is a major source of trauma associated morbidity and mortality which is now a global public health problem [1-6]. More than 14,000 people lose their lives daily due to trauma. Injury is the single largest cause of death and disability in persons below 45 years of age [7-9]. Gunshot injury (GSI) is the second most common cause of injury related deaths in the United States of America (USA) causing about 29,569 deaths in 2006 [10-12].

In the West African sub-region, early cases of significant incidences of gunshot injuries have been traced to the Nigerian civil war of 1967 to 1970 which was associated with proliferation of arms that fell into wrong hands with the aftermath of the war consequently experiencing significant increase in firearms related crimes [13-15]. Since then, there has been a rapid rise in the incidences of firearms injuries and deaths in civilian populations in sub-Saharan Africa occasioned by factors like armed struggles/agitations, poverty and its attendant increase in firearms related crimes and armed robbery, religious riots, banditry, farmers/herders clashes, political violence, etc. [4,13,16-18].

The young able bodied and productive males are often affected leading to morbidities and mortalities that have significant negative impact on societal economic productivity [16]. In addition, the human and material resources needed for the successful management of these injuries are enormous and constitute a significant drain on societal resources thus also negatively affecting the economy and the resources available for the treatment of other ailments. [5,6,12,17,19,20].

Weapons of various caliber are used in these shootings depending on their availability, the aim and circumstances surrounding such shooting. The wounding capacities of these weapons largely depend on various factors including velocity of the weapon, shape, size, eccentric movements of the bullet, its trajectory, deformation and fragmentation, range of shooting and characteristics of the affected tissue and other factors [1,21].

The aim of this study is to share our experience on gunshot injuries in our health facility and discuss our findings in the context of previous reports in the literature highlighting areas of similarities and differences with probable explanations.

## Methods

**Study design:** this was a retrospective analysis (cross-sectional study) of cases of gunshot injuries managed over a period of 3 years at the State University teaching Hospital, Oghara, Delta State. Nigeria.

**Setting/participants:** the study was carried out at the Delta State University Teaching Hospital Oghara, Delta State, Nigeria. The facility is 220 bedded multi-disciplinary centre for training medical students and resident doctors. All gunshot injury patients seen and managed at the facility over a period of 3 years (January 2016 to December 2018) were retrospectively studied.

**Data sources/measurements and variables:** case notes, ward and theatre records of all gunshot injury patients who were managed during the study period were retrieved and relevant information including: biodata, circumstances surrounding shooting, type/caliber of gun used, shooting range, number of times shot, part of the body injured, time of the day the shooting took place, injuries sustained, treatment offered, length of hospital stay and outcome of treatment were obtained and entered into a proforma designed for that purpose. Patients whose records were not complete or whose case notes could not be found were excluded from the study.

**Statistical methods/quantitative variables:** data were collated and analysed with IBM SPSS version 21, and presented in form of tables, charts, ratios and percentages. Mean, median and standard deviation were used for quantitative variable (age). Frequency and percentages were used for qualitative variables.

**Study size/bias:** being a retrospective hospital-based study the sample size is limited to the number of gunshot injured patients seen at the health facility during the 3 years study period. In addition, information obtainable were limited to what is documented in patients' records. The study may therefore not represent the actual prevalence of gunshot injuries in the area studied and results may not be conveniently generalisable.

**Funding:** this work was solely funded through contributions from authors from our earnings as medical practitioners in our hospital. No fund was received from any external source.

## Results

**Participants/descriptive data:** there were 41 cases of gunshot injuries made up of 37 males and 4 females giving a M: F ratio of 9.25: 1. The mean age of the patients was 32+9.2 years (median age =31 years) and their ages ranged from 19 to 55 years. Twenty patients (48.8%) were single, 17 (41.5%) were married and 4 (9.85%) widowed. More of the patients fell within the age group of 30-39 years (39%), followed by 29.3% of patients who fell within the age group of 20- 29 years (Table 1). Students were the most commonly affected (21%), followed by traders (Table 1).

**Table 1:** socio-demographic characteristics of patients (N = 41)

Characteristic	Frequency	Percentage
**Sex**		
Male	37	90%
Female	4	10%
**Age distribution of patients**		
10 - 19 Years	5	12.2%
20 - 29 Years	12	29.3%
30 - 39 Years	16	39.0%
40 - 49 Years	7	17%
50 - 59 Years	1	2.4%
**Marital status**		
Married	17	41.5%
Single	20	48.8%
Widowed/ divorced	4	9.8%
**Occupation of patients**		
Schooling	9	22.0%
Traders	8	19.5%
Unskilled labour	4	9.8%
Police/armed forces	4	9.8%
Driver/rider	3	7.3%
Private security	3	7.3%
Artisans	3	7.3%
Civil servants	3	7.3%
Farmer	1	2.4%
Unemployed	3	7.3%

**Figure 1:**
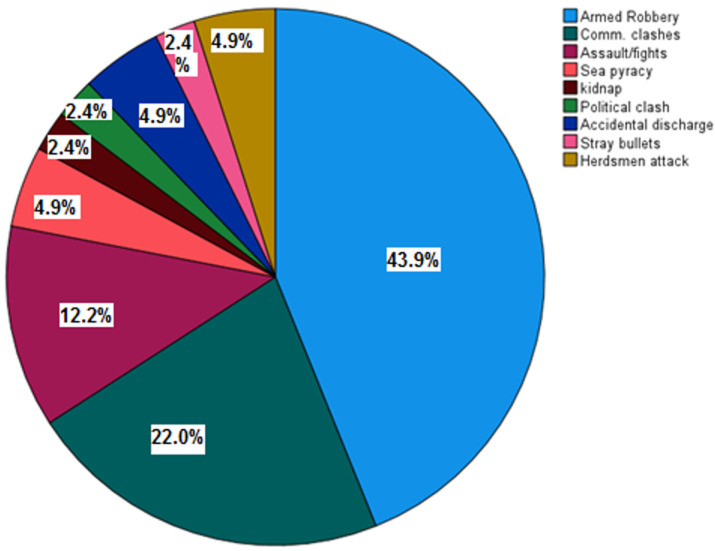
aetiologies of GSI

**Outcome data/main results:** twenty-nine patients (70.9%) were stable at presentation, while the rest (29.3%) presented in various stages of hypovolaemia/shock. Most patients (43.9%) were shot during armed robbery operations followed by 9 (22.0%) who were shot during communal clashes (Figure 1). More than half of the patients (22 = 53.7%) were shot at close range (<3m); 15(36.6%) were shot at distances greater than 3 metres while the rest 4 patients (9.8%) were shot at unknown distances.

Thirty-two patients (78%) sustained a single shot, 6 patients (14.6%) were shot twice and the rest 3 sustained multiple shots. Low velocity guns were used in 19 cases (46%), high velocity rifles in 6 cases and the weapons used in the rest 16 patients were not known. Most of the patients (61.0%) were shot in the night hours (7pm to 7am) and the rest 39% were shot during the day (7am to 7pm).

Twenty-six patients (63.4%), sustained fractures to 31 bones in addition to soft tissue injuries while, the rest 15 patients sustained only soft tissue injuries. The femur was the most frequently fractured bone in 7 patients (17.1%) followed by the ulna in 5 patients (12.2%) Table 2.

**Table 2:** fractures from GSI (31 fractures in 26 patients)

Bones	Frequency	Percentage
Femur	7	22.6%
Ulna	5	16.1%
Radius	4	12.9%
Hemerus	3	9.7%
Hand bones	4	12.9%
Tibia	3	9.7%
Fibula	2	6.5%
Rib	1	3.2%
Skull	1	3.2%
Facial bone	1	3.2%
	31	100.0%

The extremities were the most frequently injured part of the body in 27 patients (65.9%), abdomen/pelvis in 16 patients, chest in 6 patients and head/neck region in 1 patient. Some patients sustained injuries to more than one body part leading to 50 injuries in the 41 patients.

Nineteen patients (46.3%) were brought to the emergency room (ER) by relatives, 13 (31.7%) by passersby, 8 (19.5%) by security agencies and 1 (2.4%) came to the ER by himself.

Fifty-three point seven per cent (22) of the patients were brought to the ER directly from the scene of shooting, 43.9% (18) had one form of treatment or the other in a peripheral hospital before being brought to the ER while 1 was initially treated in a patent medicine store before coming. Treatments offered included: initial wound debridement in 32 patients (78%), exploratory laparotomies in 11 patients who sustained abdominopelvic injuries (Table 3). The average length of hospital stay for the patients was 19.8+24 days, range 1 to 150 days. On the average patients who had fractures tended to stay longer in the hospital.

**Table 3:** procedures done for GSI patients

Procedures	No. of patients
Debridement	32
Cast/slab application	30
Exploratory laparotomies	11
Tube thoracotomies	5
External fixation	10
Orif	4
Craniotomy	1
Limb amputation	2
Fasciotomies for compartment syndrome	2
Flap coverage of wounds	2
Split skin grafts	3
**Total**	**102**

Twenty-five patients (61%) were treated successfully and discharged; eleven patients (26.8%) discharged themselves against medical advice after initial resuscitation; 3 died and 2 were referred to other health facilities as a result of doctors' strike. Out of the 25 patients treated and discharged, 9 had some residual functional deficits at the time of discharge, including 2 amputees.

## Discussion

The incidence of gunshot injuries (GSI) in civilian populations is on the rise worldwide with its attendant morbidity and mortality especially among the young and productive age groups in the society who are the ones most frequently affected. GSI has thus, become a global public health problem and a therapeutic challenge to surgeons [2,5,6,11,18,20,22-26]. Forty-one patients had GSI to various parts of the body over a period of 3 years in our study. This was made up of 37 males and 4 females giving a male: female ratio of 9.25: 1. The finding of a large male preponderance in this study has been the general trend in the literature over the years [2,6,13,15,16,18,22,24,27-29]. The reasons for this trend have been attributed by many authors to the fact that males are usually the bread winners, take more risks, are more outgoing and thus more involved in the activities that predispose to GSI including wars, armed robbery, communal clashes, political thuggery etc. [15,18,22].

The most active and economically productive age groups in the society are often the ones largely affected by GSI thus, dealing a deadliest blow on societal economic productivity in addition to massive consumption of the health resources of the society in form of medical care for the victims some of which end up with lifelong disabilities. In this study, the most affected persons (68%) were between ages 20 and 39 years. This finding is similar to what has been reported in several previous studies although percentages vary from study to study [5,16,18,22,23,26,29,30]. Government at all levels and all stake holders must therefore, make concerted efforts to reduce the incidence of GSI and mitigate its adverse effect on economic productivity and other economic and health indices.

The mean age of patients in this study was about 32 years. Similar means of 32.5 years, 32.2 years and 31.7 years have been reported by Omoke *et al*. [1], Umaru *et al*. [4] and Bodalal *et al*. [30] respectively. It is however, higher than the means reported by other authors [6,13,15,22,28] while it is lower than that reported by yet another group of authors [2,16]. Students and traders were the most frequently affected by GSI in this study. Similar findings have been reported by other authors [4,16,18,22,26]. This might be attributed to the fact that these group of persons frequently travel on the roads where most of the shootings by armed robbers take place. Oboirien *et al*. [24] from Sokoto in Nigeria however, reported that farmers were the most frequently affected by GSI in their study, an observation they attributed to the problem of frequent farmers/herders clashes in that region.

Most GSI victims in our study were shot during armed robbery operations. The scenario is the same in many places in Nigeria as observed in studies from many other parts of the country [1,2,4,13,16-18,22,24]. However, Udosen *et al*. [14] from Calabar also in Nigeria reported accidental discharge and stray bullets as the commonest motives for shooting in their study, while Umaru *et al*. [26] in a 2011 study from Maiduguri North-east Nigeria, reported that most of the victims of GSI in their study were casualties of Boko Haram insurgents. In contrast, studies by Singh *et al*. [5] from India and Norton *et al*. [28] from UK, reported homicidal assault as the main motive behind gunshot injuries in their studies. The dominant role played by armed robbery as a major aetiological factor in gunshot injuries in Nigeria - a role which seems to be increasing by the day - has been largely attributed to poverty, deprivation, high rate of unemployment and proliferation of small arms and ammunitions due to poor governance, activities of insurgents and unscrupulous politicians who arm political tugs during elections [24].

Low velocity weapons were largely used in inflicting injuries on victims in this study - most of these weapons being the type locally fabricated. Similar findings have been reported by some previous authors [1,13,15,18,24,31,32]. In contrast, high velocity weapons have been reported to be the most frequently used in other studies [2,26,30]. Various factors may be responsible for the caliber of gun used in inflicting injuries on victims in an environment including prevailing social circumstances. It is usual to expect the preponderance of low velocity weapon usage in studies in civilian populations. However, situations such as the recent upsurge in insurgency in North Eastern Nigeria has led to the predominance of the high velocity/high caliber weapons in civilian populations outside a conventional war situation as reported by Umaru *et al*. [26] in a 2011 study from Maiduguri. This contrasted with the observation of the preponderance of the usage of low velocity weapons in an earlier study from the same environment [4].

Over 60% of GSI victims in this study were shot during the night hours. This agrees with observations in many previous studies in our clime [1,22,33-35]. The reason for this is not farfetched. Armed robbers and other criminal elements in the society are usually more comfortable perpetrating their trade under the cover of the darkness of the night. In contrast however, Iloh *et al*. [36] from Umuahia in Nigeria, reported that most gunshot injuries in their study occurred during the day. The armed robbery was also reported as the commonest motive for shooting in their study. Their explanation for this unusual finding was that armed robbers and other criminal elements were now becoming more daring and were no longer afraid of day light when carrying out their evil trade.

The amount of energy dissipated into tissues by bullets and thus the degree of tissue destruction most times depend amongst other factors on the range of shooting-the closer the range many times, the greater the tissue destruction and vice versa especially for low velocity weapons. More than half of the gunshot victims in this study were shot at close range. This is similar to observations reported by Lasebikan *et al*. [2] and Oboirien *et al*. [24] from Enugu and Sokoto respectively in Nigeria. The site of injury/organ injured are also very important determinants of morbidity and mortality. Head and neck injuries are usually more deadly followed by trunk injuries compared with injuries to the extremities [17,26,28,37,38]. Of the 3 patients that died in this study, 1 sustained a depressed skull fracture while the other 2 sustained chest/abdominal injuries requiring tube thoracostomy, laparotomies, colostomies and ICU admissions.

Furthermore, in our study and many others from different areas in Nigeria [1,4,8,15,17,18,26,28-30], the extremities (upper and lower limbs) have been observed to be the most frequently affected in GSI victims compared with other parts of the body. The reason for this finding has been largely attributed to the fact that the aim of shooting by armed robbers and kidnappers in our environment most times is to demobilize their victims and not to kill [15,22,24]. In contrast, reports from Indian GSI studies by Singh *et al*. [5] and Khan *et al*. [12] showed that the trunk and head and neck regions were more affected by GSIs respectively. Abdominal injuries have also been reported as the most common type of GSI in Benin City, Nigeria, in a study by Okobia and Osime [39].

Fractures associated with GSI occurred in about 63% of cases in the study, with the lower limb being the most frequently affected here-precisely the femur in this series. Umaru *et al*. [26], similarly reported femur as the most frequently fractured in their study. However, the tibia and the fibula have been reported as the most frequently fractured bones resulting from gunshots by some other authors [6,16]. This finding of lower limb fractures further confirms the reasoning that most times armed robbers and other criminal elements in our environment shoot their victims in order to demobilize rather than kill them [2,15,16,22,24].

Gunshot injuries are an important cause of morbidity and mortality in our environment. Unfortunately, gunshot victims who die at the scene of shooting and those who die before getting to the hospital including those treated by traditional bone setters and other peripheral facilities are largely unaccounted for due to poor documentation or poor data handling in our clime. About 7% of the GSI patients in this study died. This percentage is higher than the 1.7%, 1.8% and 3.9% mortalities recorded by Abbas *et al*. [22], Lasebikan *et al*. [2] and Singh *et al*. [5] respectively. Higher percentage mortalities of; 10.5%, 12.5% and 16.5% - have however been reported by Aigoro *et al*. [15], Mohammed *et al*. [18] and Solagberu *et al*. [13] respectively from their various studies. The severity and outcome of injuries are most times the result of a combination of factors which may include the velocity/caliber of weapon used, the range of shooting, site/ organ injured, age and pre-morbid health of victim, pre-hospital care and care received in the hospital among other factors [16,18,28,37,38].

**Limitations:** the limitation of this study included that it is hospital-based and might not be a true reflection of the prevalence and epidemiology of gunshot injuries in the general population. Many patients with gunshot injuries may never come to orthodox hospitals for treatment especially armed robbers and other criminal elements for fear of being apprehended and made to face the law. In Nigeria gunshot injuries are by law reported to the police by managing health facilities. Another limitation is that which apply to all retrospective studies as data are limited to what was recorded for each patient.

## Conclusion

From the foregoing discussion, the socioeconomic impact of GSI in Nigeria cannot be underestimated. The incidence of GSI has become worse with the continuous down turn of our economy and the accompanying increase in socioeconomic hardship among the citizenry. Armed robbery, kidnapping, political thuggery, banditry, insurgency, herders/farmers clashes and other criminal activities are as a result on the increase with associated geometric increase in GSI incidence. All hands must therefore, be on deck to curb the burden of GSI by adopting appropriate strategies to fight armed robbery and other criminal activities in our society. Our law enforcement agencies must be constantly equipped with the needed material and human resources to effectively fight crime. At the same time, government at all levels must strive to improve on the socioeconomic conditions of the people, especially the youths through job creation and other forms of economic empowerment.

### What is known about this topic

- Incidence of gunshot injuries is increasing worldwide;
- Gunshot is a significant cause of trauma;
- Reasons/aetiology for shooting vary from study to study depending on various factors.

### What this study adds

- Armed robbery ranked the most common motive for shooting in GSI victims in the Niger Delta region of Nigeria;
- Poor or non-existent pre-hospital care for GSI victims in the region of study;
- This study further corroborates the observation that young active males are the ones mostly affected by GSI.
